# Severe proptosis during cataract surgery

**DOI:** 10.1016/j.ajoc.2023.101901

**Published:** 2023-07-20

**Authors:** Lauren Ton, Wanlin Zhang, Murtaza Saifee, Anushka Walia, Julius Oatts, Ying Han

**Affiliations:** a School of Medicine, University of California San Francisco, San Francisco, CA, USA; b University of California Berkeley, Berkeley, CA, USA; c Department of Ophthalmology, University of California San Francisco, San Francisco, CA, USA

**Keywords:** Proptosis, Glaucoma drainage device, Phacoemulsification

## Abstract

**Purpose:**

We report an unusual case of severe proptosis during phacoemulsification in a 58-year-old female with a history of Crohn's disease, bilateral chronic panuveitis, prior bilateral central retinal vein occlusion, and uncontrolled steroid-associated ocular hypertension requiring bilateral Ahmed glaucoma drainage device (GDD) implantation with pars plana tube placement.

**Observations:**

During phacoemulsification of the right eye, the patient developed significant proptosis. Following lid speculum removal and mechanical eyelid manipulation, the proptosis resolved within 20 minutes without requiring a lateral canthotomy. The patient had no permanent visual complications.

**Conclusions and Importance:**

The likely pathophysiology of intraoperative proptosis in this case was accumulation of fluid in the retrobulbar space due to a functioning Ahmed tube shunt with the tube placed in the vitreous cavity. To avoid this complication, concurrent cataract surgery may be considered for patients with pars plana tube placement GDD surgery.

## 1 Introduction

Proptosis, the anterior displacement of the eye from the orbit, can result from a wide variety of pathologies including autoimmune disorders, trauma, neoplasms, infections, and orbital vascular disorders.^1^ Causes of acute onset unilateral proptosis include orbital cellulitis, intraorbital neoplasms, retrobulbar hemorrhage, or cavernous sinus thrombosis. Severe proptosis may lead to corneal abrasions and optic nerve damage caused by stretching, resulting in transient or permanent visual impairment. Intraoperative proptosis is a rare but serious complication of phacoemulsification surgery. Although there have been reports of acute proptosis developing during phacoemulsification, these cases are almost exclusively due to retrobulbar hemorrhage. In this case report, we present an unusual case of acute unilateral proptosis during phacoemulsification in a patient who had undergone Ahmed glaucoma valve (AGV) implantation with tube placement in the vitreous cavity.

## 2 Case report

A 58-year-old female with a history of Crohn's disease, bilateral chronic non-granulomatous panuveitis, prior bilateral central retinal vein occlusion with cystoid macular edema presented for management of medically uncontrolled bilateral steroid-associated ocular hypertension. She underwent uncomplicated bilateral sequential GDD implantation (Ahmed FP7; New World Medical, Rancho Cucamonga, California). Given her active uveitis and significant vitreous debris, the decision was made to combine AGV implantation with pars plana vitrectomy and Retisert implantation (Bausch + Lomb, Rochester, New York). The Ahmed tubes were placed in the pars plana given extensive peripheral anterior synechiae in both eyes. Additionally, cataract surgery was not performed concurrently to minimize post-operative inflammation given her active inflammation at the time of surgery. Seven months later, she underwent uncomplicated phacoemulsification in the left eye with an unremarkable post-operative course. Two months later, the decision was made to perform cataract surgery on the right eye. At that time, her examination showed best corrected visual acuity (BCVA) of counting fingers at 1 foot in the right eye and 20/200 in the left eye. Intraocular pressure (IOP) was 12.5 mmHg and 10 mmHg in the right and left eye, respectively. On slit lamp examination, the patient had no active intraocular inflammation and 3+ nuclear sclerosis in the right eye.

**Fig. 1:**
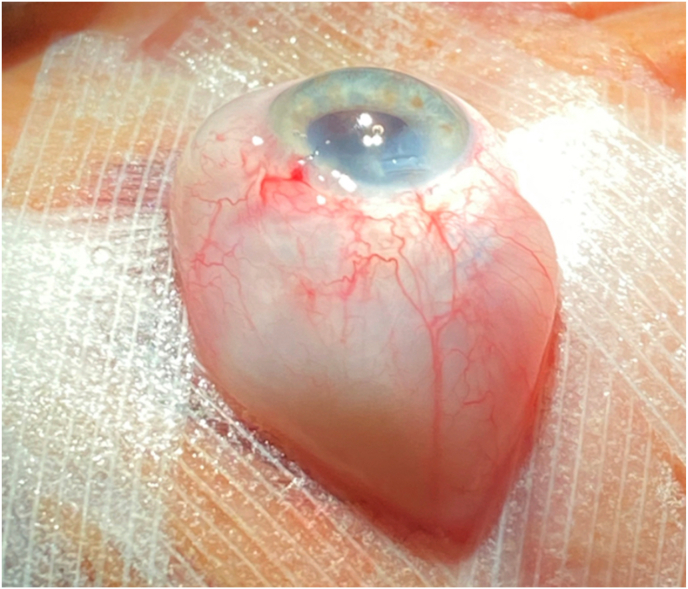
Significant intraoperative proptosis of the right eye noted following nucleus removal.

Phacoemulsification with intraocular lens implantation of the right eye was performed under general anesthesia due to a history of chronic cough. Standard phacoemulsification was performed through a temporal clear corneal incision and the nucleus was removed using a non-stop chop technique. During irrigation and aspiration, the patient developed significant proptosis of the operative eye (Fig. 1). At this time, the anterior chamber was maintained, though there was some mild temporal iris prolapse through the main wound. Cortical removal was completed and the capsular bag was filled with viscoelastic (ProVisc; Alcon, Fort Worth, Texas) and a 1-piece intraocular lens was inserted into the capsular bag. In order to avoid anterior chamber shallowing, the remaining viscoelastic material was not removed. The paracentesis and the main wound were both closed with a single 10–0 nylon suture. The eyelid speculum was removed. Throughout this time, the eye remained soft with no evidence of elevated IOP. Indirect ophthalmoscopy was performed revealing a pale optic nerve with no new retinal or choroidal pathology.

**Fig. 2:**
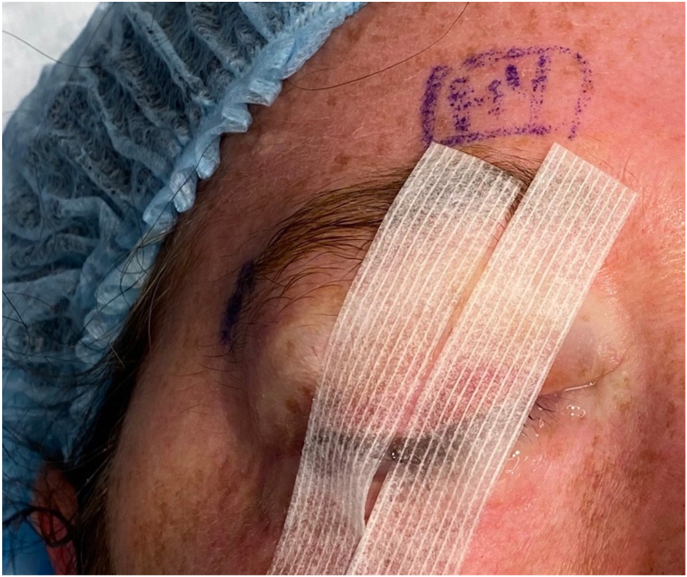
Within 20 minutes of surgery completion and eyelid speculum removal, the patient's proptosis had resolved, eliminating the need for a lateral canthotomy.

**Fig. 3:**
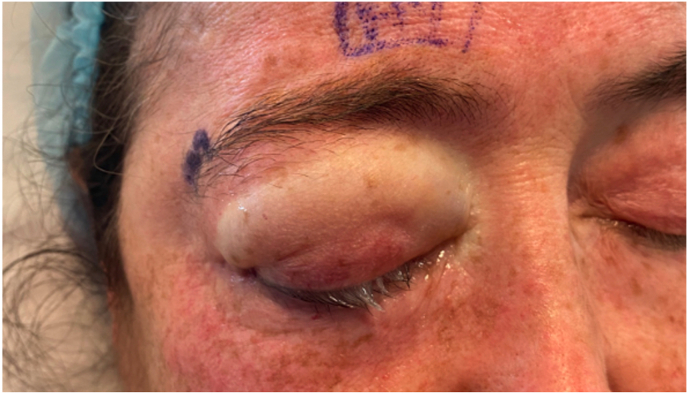
Immediately after surgery, the patient was noted to have edema of the repositioned upper and lower eyelids.

The oculoplastics team was immediately consulted. The patient's clinical presentation was not consistent with retrobulbar hemorrhage, as the eye remained soft without significant posterior pressure. Either mechanical manipulation of the eyelids or lateral canthotomy was recommended. The proptosis had improved significantly within 20 minutes of eyelid speculum removal. Thus, the upper and lower eyelids were able to be mechanically repositioned, eliminating the need to perform a canthotomy. The eyelids were able to close, though the patient had marked eyelid edema (Fig. 2, Fig. 3).

**Fig. 4:**
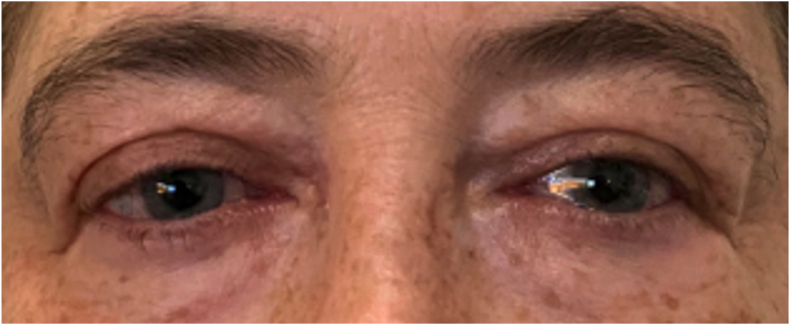
On postoperative day 1, the patient had mild chemosis with complete resolution of proptosis.

On post-operative day 1, the patient's visual acuity in the right eye was 20/200 with an IOP of 14 mmHg (Fig. 4). After 1 week, the visual acuity had improved to 20/70, which was her expected vision given a history of central retinal vein occlusion. At one month post-operatively, her BCVA was 20/60.

## 3 Discussion

The phenomenon of intraoperative proptosis during phacoemulsification in a patient with a prior GDD has not been previously reported. Phacoemulsification in patients with functioning GDDs is common, but proptosis is an exceedingly rare complication. While proptosis during phacoemulsification may be indicative of retrobulbar hemorrhage, the patient's clinical presentation was not consistent with this etiology. We propose another possible cause for this intraoperative complication. Proptosis may have been the result of fluid accumulating in the retrobulbar space due to the patient's functioning AGV with tube placed in the vitreous cavity. Intraoperatively, 300 mL of irrigation fluid was used, and it is likely that some of this migrated from the anterior chamber into the vitreous space. From there, the fluid could flow through the patent AGV tube to accumulate in the sub-Tenon and retrobulbar spaces. Given that the retrobulbar space is fixed and surrounded by bone, fluid accumulation will cause forward displacement of the eye, leading to proptosis.

There is limited literature on proptosis during phacoemulsification surgery after GDD implantation. Rowland et al. described the only reported case of extreme globe subluxation during phacoemulsification in a patient with a Baerveldt Glaucoma Implant with tube in the anterior chamber.^2^ The affected eye had dramatically elevated IOP and shallowing of the anterior chamber, requiring mannitol and pars plana vitrectomy. This differs from our patient's presentation, in which the IOP was not elevated and the anterior chamber was well-maintained despite proptosis.

The pars plana tube placement in our patient differed from the more common anterior chamber tube placement and may explain why proptosis developed in this case, while rarely occurring in patients with prior GDD surgery. For patients with tube placement in the anterior chamber, the viscoelastic injected into the anterior chamber during phacoemulsification likely occludes the tube or decreases the rate of tube drainage. This limits the efflux of fluid into sub-Tenon's and retrobulbar spaces. However, a tube in the vitreous cavity after vitrectomy may cause unobstructed egress of irrigation fluid, increasing the risk of proptosis. Notably, the patient underwent phacoemulsification without complications in her contralateral eye with similar AGV and tube placement in the vitreous cavity. However, the tube in the contralateral eye was placed in close proximity to the posterior capsule. Thus, it is possible that the posterior capsule acted as a membrane to occlude the tube during phacoemulsification, analogous to viscoelastic limiting fluid conductance via an anterior chamber tube. All these hypotheses may explain the rarity of intraoperative proptosis in patients with functional GDD.

Additionally, other factors may contribute to the likelihood of intraoperative proptosis in a patient with functional GDD, including relative inexperience of a surgeon, longer duration of surgery, or a larger volume of fluid used during the surgery.

Correction of intraoperative proptosis was prioritized to avoid compression and stretch of the optic nerve, which can cause optic nerve defects and vision loss.^3^ Fortunately, in this case, the proptosis self-resolved within 20 minutes of completing the procedure when irrigation fluid was stopped. Lateral canthotomy may have been required to prevent optic neuropathy if the condition had not resolved spontaneously.

## 4 Conclusion

In conclusion, we report the first case of intraoperative proptosis during phacoemulsification occurring as a complication of a GDD with tube present in the vitreous cavity. Irrigation fluid drained from the vitreous cavity into the retrobulbar space via the tube, resulting in forward displacement of the eye. With cessation of irrigation, proptosis self-resolved within a few minutes. This case highlights the importance of awareness of the risk of proptosis during phacoemulsification in patients with vitreous GDD tube placement. To avoid this complication, concurrent cataract surgery may be considered at the time of GDD surgery when pars plana tube placement is planned.

## Patient consent

The patient provided oral and written consent to the publication of the case.

## Funding

No funding or grant support.

## Authorship

All authors attest that they meet current ICMJE criteria for Authorship.

## Declaration of competing interest

The authors declare that they have no known competing financial interests or personal relationships that could have appeared to influence the work reported in this paper.
